# Pediatric basic course goes virtual: transition from face to face to hybrid learning in pediatric critical care

**DOI:** 10.1186/s13052-023-01461-4

**Published:** 2023-06-07

**Authors:** Anna Zanin, Angela Aramburo Caragol, Luca Tortorolo, Michele Patui, Beatrice Pedrini, Joe Brierley, Bruce Lister, Paola Cogo

**Affiliations:** 1 Department of Women’s and Children’s Health, University of Padua, via Giustiniani 3, Padova, 35100 Italy; 2 Pediatric Intensive Care Unit, Royal Brompton Hospital NHS Foundation Trust, London, UK; 3 Terapia Intensiva Pediatrica del Policlinico Universitario Agostino Gemelli IRCCS, Roma, Italy; 4 Department of Medicine DAME-Division of Pediatrics, University of Udine, Udine, Italy; 5 Critical Care Units, Great Ormond Street Hospital, Great Ormond Street, London, UK; 6 College of Intensive Care Medicine of Australia and New Zealand, Victoria, Australia

**Keywords:** Pediatric critical care, Learning, Education, Pediatric BASIC course, Training program, ESPNIC

## Abstract

**Background:**

To explore the impact of the transition from a traditional face-to-face course delivering essential contents in pediatric critical care to a hybrid format consisting of an online pre-course self-directed learning, an online facilitated discussion, and a face-to-face edition.

**Methods:**

Attendees and faculty were surveyed after the face-to-face course and the hybrid version to evaluate the effectiveness and satisfaction of participants with the course.

**Results:**

Fifty-seven students attended multiple formats of the Pediatric Basic Course between January 2020 and October 2021 in Udine, Italy. We compared course evaluation data from the 29 attendees of the face-to-face course with the 28 of the hybrid edition. Data collected included participant demographics, participant self-assessed pre and post-course ‘‘confidence’’ with a range of pediatric intensive care-related activities, and their satisfaction with elements of the course. There were no statistical differences in participant demographics or pre and post-course confidence scores. Overall satisfaction with the face-to-face course was marginally higher, 4.59 vs. 4.25/5, but did not reach significance. Pre-recorded lectures which could be viewed several times, were highlighted as a positive for the hybrid course. Residents found no significant differences comparing the two courses in rating the lectures and the technical skills stations. Hybrid course facilities (online platform and uploaded material) were reported to be clear, accessible, and valuable by 87% of attendees. After six months, they still find the course relevant to their clinical practice (75%). Candidates considered the respiratory failure and mechanical ventilation modules the most relevant modules.

**Conclusions:**

The Pediatric Basic Course helps residents strengthen their learning and identify areas to improve their knowledge. Both face-to-face and hybrid model versions of the course improved attendees’ knowledge and perceived confidence in managing the critically ill child.

**Supplementary Information:**

The online version contains supplementary material available at 10.1186/s13052-023-01461-4.

## Background

In Pediatric Critical Care Medicine (PCCM), the need to learn and maintain clinical skills is crucial. However, this can be challenging due to the complex and rapidly changing nature of clinical practice, the relatively unstable make-up of the clinical interprofessional and multidisciplinary teams, a heavy reliance on changing technology, and the high acuity of children’s critical illness [1, 2]. For these reasons, the ongoing education, assessment, and maintenance of skills required by the health professionals who care for critically- ill children is a priority [1, 2]. Moreover, because of the increasing number of trainees and changes in medical rotas, any individual trainee has reduced access to procedures, so it is vital to proactively develop bespoke training opportunities for those developing or maintaining their skills [3, 4].

There are few countries with a structured and organized training, certification, and revalidation system for PCCM [1]. The Accreditation Council for Graduate Medical Education and the American Board of Pediatrics assume this responsibility in the US; some European countries have similar individual national structures, but the European landscape of PCCM education is highly variable. In Italy, there is no standardized PCCM training program incorporating residency and fellowship programs, no certification oversight, and no specialty certification maintenance. Resident learning occurs mainly through direct patient care during clinical rotations. Few residency programs offer a structured rotation in a tertiary PICU.

Moreover critically ill children can be managed in the PICU by pediatricians, anesthesiologists and neonatologist-led NICUs admitting only critically ill infants [5].

Pediatric BASIC consists of two days of lectures, simulated illness and injury scenarios, procedural skill stations, and discussion groups. The course, run globally since 2012, specifically targets pediatric trainees, adult emergency room physicians (whether ED, ICU, or anesthesiology), and all members of the pediatric intensive care interprofessional healthcare team. The course ensures that all candidates receive high-quality, individualized support in simulation and practical ICU skills. However, this same quality means the BASIC approach was especially vulnerable to the effects of the SARS-COV-2 pandemic due to social distancing and travel restrictions.

Pediatric BASIC has only recently been established in Italy by local Italian pediatric intensive care fellows and specialists supported by an international faculty of pediatric intensive care specialists. This course was first delivered in Italy at the University of Udine in collaboration with and supported by the European Society of Pediatric and Neonatal Intensive Care (ESPNIC). Pediatric residents, pediatric and adult emergency trainees, adult ICU-anesthesia trainees/specialists, and experienced PICU and emergency medicine nurses have participated.

The inaugural course in January 2020 was delivered using the conventional face-to-face format, which included lectures and practical simulation (procedural skills station and scenarios) for three days. After the beginning of the pandemic, the transition to online classes and the cancellation of clinical practice sessions and rotations negatively impacted all medical education fields despite increasing clinical demand [6]. As many courses and congresses were canceled or postponed, academic institutions and scientific societies have proposed and applied virtual formats as the “new normal” for postgraduate and continuing medical education.

In this sense, the COVID-19 pandemic represented a true accelerator of existing teaching evolution to remote learning [6]. The local Italian and international faculty developed a virtual platform in collaboration with the University of Udine, which hosted pre-recorded lectures covering various topics from the traditional face-to-face format course. This material was available to the candidates six weeks before the hybrid course, supported by the availability of the faculty every 15 days to answer questions and clarify points of uncertainty. We provided fifteen 30-minute lectures covering the most important topics in PCCM recorded by an international pool of experts from Australia, Europe, and the US who already teach standard BASIC courses. All lectures were specially recorded for this edition of the BASIC course; each provided insights on specific subtopics according to the program of the course, with some clinical cases, presented.

**Fig. 1:**
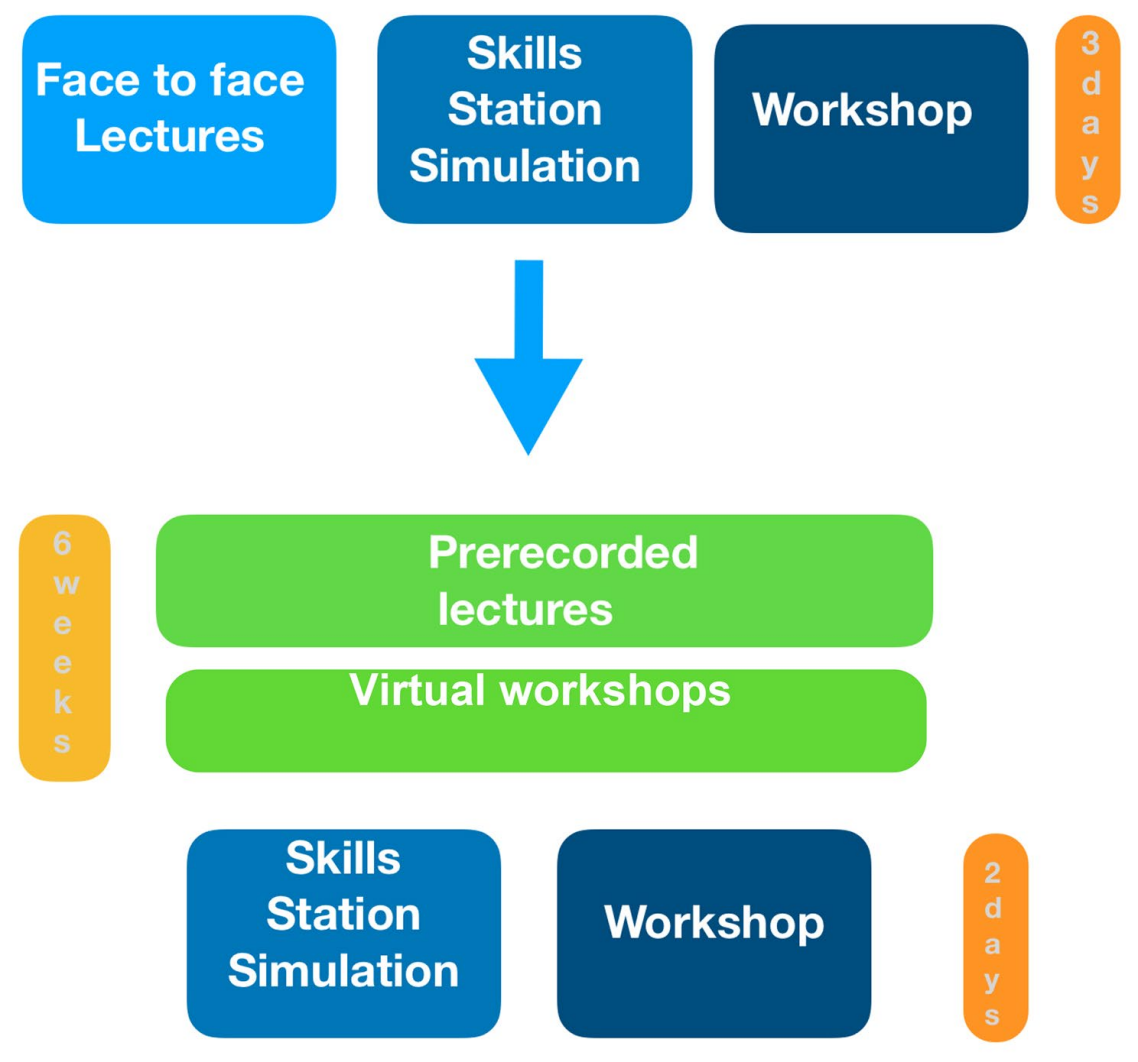
Structure of the Pediatric Basic course before and after the online transition

The two-day face-to-face practical session was conducted after four days of virtual workshops with remote simulation and small group discussions on selected topics (Fig. 1).

This hybrid training format was delivered twice in Udine (Italy). This study aimed to assess the feasibility of the hybrid model and evaluate the course’s quality in terms of the outcome of assessment (pre and post-course MCQ test) and satisfaction by both trainees and instructors.

## Methods

The study was approved by the University of Udine institutional review board (University of Udine). We performed a retrospective analysis of prospectively collected data from the pre and post-survey delivered to all the attendees who participated in the first face-to-face course in January 2020 and in the hybrid format delivered between January and December 2021. All participants enrolled in the course spontaneously, which had been promoted and advertised by the University of Udine, the BASIC course website, and the ESPNIC website.

The list of topics covered is shown in Table 1. To obtain course completion certification, candidates had to take a pre-course multiple-choiceand pass both a post-course multiple-choice test and an ongoing performance assessment. Data collected included participant demographics, pre and post-assessment scores (MCQ) dealing with the topics covered during the course and a survey of overall satisfaction with the course, instructors’ evaluation, and pre and post-course confidence levels in participants’ ability to perform a range of skills and activities related to PCCM.

**Table 1:** List of topics of Pediatric BASIC course

Day 1	Day 2
**Lectures**	**Lectures**
Teamwork and Crisis Resource Management	Fluids & Shock
Assessment of the Seriously Ill Child	Severe Sepsis & Septic Shock
Acute Respiratory Failure	Trauma
Airway Management	Traumatic Brain Injury
Mechanical Ventilation – Basics & Modes	Pediatric Neurological Emergencies
Mechanical Ventilation – Settings	Acute Kidney Injury
Cardiopulmonary Interactions	Nutrition
Congenital Heart Disease	Transport Exercise
Physiological Monitoring	
**Skill Stations**	**Skill Stations**
Resuscitation	Mechanical Ventilation 3
Airway	Assessment & Shock
Acid Base	Trauma
Mechanical Ventilation 1	Vascular Access
Mechanical Ventilation 2	Analgesia & Sedation

Before attending the Pediatric BASIC Course, candidates undertook an online multiple-choice test. The test scores helped guide Instructors on the level at which to pitch the course.

The participants took a different MCQ test at the end of the course to assess their knowledge of the course content and ability to apply it in a clinical context. Doctors and nurses completed the same pre and post-course MCQ. All participants completed both the pre and post-course MCQ.

Candidates were also asked to anonymously complete two identical survey forms at the beginning and end of the course to help evaluate the impact of the course. The candidates were asked to state how much they agreed with a positive statement about being confident within a particular clinical skill, choosing from a range of options from strongly agree (5 points) to strongly disagree (1 point). The surveys were then collated, and an average score was determined for each question using the points scale.

Survey items were measured on a 5-point Likert scale. Dichotomous outcome measures were generated by combining the “Agree” and “Strongly Agree” into one category and comparing this to the other categories. Categorical data were analyzed primarily by Fisher’s exact or Chi-square tests. Data are expressed as total counts, percentages, and means (+/- SD).

## Results

Fifty-seven participants attended the Pediatric Basic Course between January 2020 and December 2021 in Udine, Italy. The topics covered in both face-to-face (FC) and hybrid courses (HC) are listed in Table 1, and the course structure before and after the transition is shown in Fig. 1. Demographic data and assessment scores of the 29 attendees of the face-to-face course and the 28 of the hybrid version are listed in Table 2. Participant demographics were homogenous, except for the hybrid group of anesthesiologists that was not present in the standard face-to-face group. Specifically, for the hybrid course, participants highlighted an appreciation of pre-recorded lectures as the material that could be viewed multiple times, and most attendees (87%) found the platform easy to use. The overall evaluation of both the FC course and the HC one was positive. In both courses, 94% of the attendees found the educational material valuable and interesting. 69% of attendees stated that the HC met their expectations compared to 72% for the FC, where 4% of participants also reported that the course exceeded expectations.

**Table 2:** Demographic characteristics of the attendees of Face to face and Hybrid course

	Face to face course n (%)	Hybrid course n (%)	p value
**Number of attendees**	29 (100)	28 (100)	
**Pediatric Residents**	14 (49)	14 (49)	
**Anesthesiologists**	0 (0)	6 (22)	
**Pediatricians**	11 (38)	2 (7)	
**PICU physicians**	1 (3)	1 (3)	
**Emergency Doctors**	1 (3)	5 (19)	
**Nurses**	2 (7)	0 (0)	
**Gender (M/F)**	14/15 (48/52)	9/19 (32/68)	
**Italy**	24 (82)	28 (100)	
**International**	5 (8)	0 (0)	
**Pre assessment score**	22.3	26.5	< 0.05
**Post assessment score**	21.13	26.8	< 0.05

**Fig. 2:**
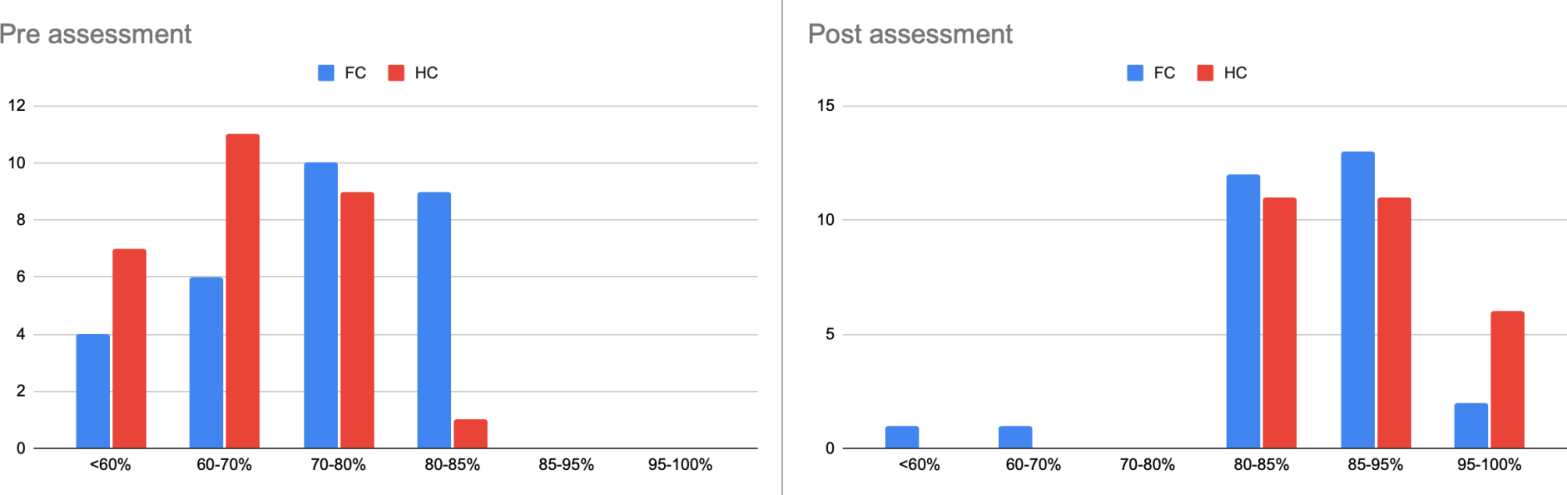
Pre and Post Post assessment scores (% of right answers) of face to face and hybrid course (FC = face to face course, HC = hybrid course) attendees

Ninety-three (27/29) of the FC participants FC and 100% of the HC participants achieved a score > 80% correct in the post-course MCQ (Fig. 2). Although the pre and post-course MCQ tests were not identical and were taken under different conditions, the questions had a similar degree of difficulty. Pre and post-assessment scores were significantly higher in the hybrid course (p < 0.05) (Table 2). There was an observed mean improvement of 4.0 (range − 1 to + 11) in the raw scores between the pre and post-assessment tests in the face-to-face course and 6.7 (range + 1 to + 12) in the hybrid course.

**Fig. 3:**
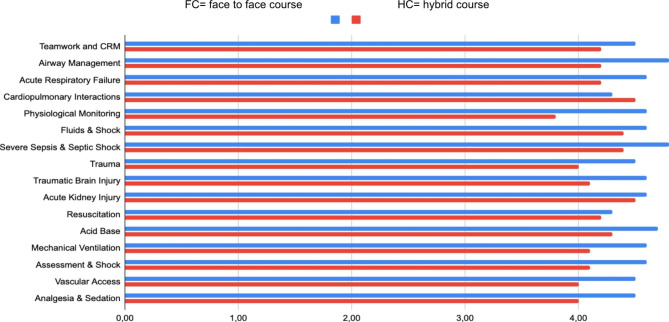
Mean value of lectures and skills station rating scales (Likert 1–5) of face to face and hybrid course (FC = face to face course, HC = hybrid course)

Twenty-three over twenty-nine (79%) candidates of the face-to-face group and 24/28 (86%) of the hybrid group returned their feedback forms at the end of Day 2. Feedback was generally very positive about the course content (Fig. 3). The overall average scores of the responses were 4,59 (+/- 0,14) for the face-to-face course and 4,22 (+/- 0,22) for the hybrid, with no significant differences as in the change in participant ‘comfort’ before and after the course (Table 3). Student feedback for the HC was as positive as for the FC; in the residential course, the more highly rated lectures were Airway Management (4,8 (+/- 0,14) and Severe Sepsis and Septic Shock (4,8 (+/- 0,13); in the hybrid course, Acute Kidney Injury (4,5 (+/- 0,13) and Cardiopulmonary Interactions (4,5 (+/- 0,19). It is worth noting that the same instructors presented these four modules in both face-to-face and hybrid courses. We also collected at the end of the courses some open comments by attendees in order to allow them to give better feedback on any difficulties or provide suggestions: among the attendees’ comments collected in a word cloud, we found that ventilation and fluids management were perceived as important topics in pediatric critical care that deserved a deeper and longer learning module. Nephrology was reported as a complicated topic that could have a broader and deeper approach. Among the suggestions we collected, thanks to the open questions, the strengths listed were that the course is run by an international faculty with the possibility to have a direct exchange in terms of answering questions and having some practical simulation and skills stations. In all courses, the attendees asked for more time dedicated to practical skills stations and scenarios. Another suggestion was to stratify attendees according to their previous experience in managing the critically ill child.

**Table 3:** The change in Participant ‘comfort’ before and after the course

	Pre-Course Survey (Average out of 5)		Post-Course Survey (Average out of 5)		p value	% of candidates indicating a shift in confidence	
	FC	HC	FC	HC		FC	HC
I am confident in assessing a seriously ill and injured child	3.0	3.3	3.8	3.9	ns	39%	6%
I am confident in diagnosing and managing respiratory failure in children	3.2	3.1	3.8	3.7	ns	25%	12%
I am confident in the management of an infant or child with congenital heart disease	2.3	2.1	2.8	3.8	ns	39%	38%
I am confident in performing practical procedures in an emergency/resuscitation situation.	2.8	3.3	3.1	4.0	ns	14%	6%
I am confident in managing the child with severe sepsis/septic shock	3.0	3.2	3.6	4.1	ns	29%	12%
I am confident managing trauma in children	3.0	3.3	3.4	4.0	ns	25%	6%
I am confident managing a child with a traumatic brain injury.	2.9	3.0	3.5	3.9	ns	25%	6%

**Fig. 4:**
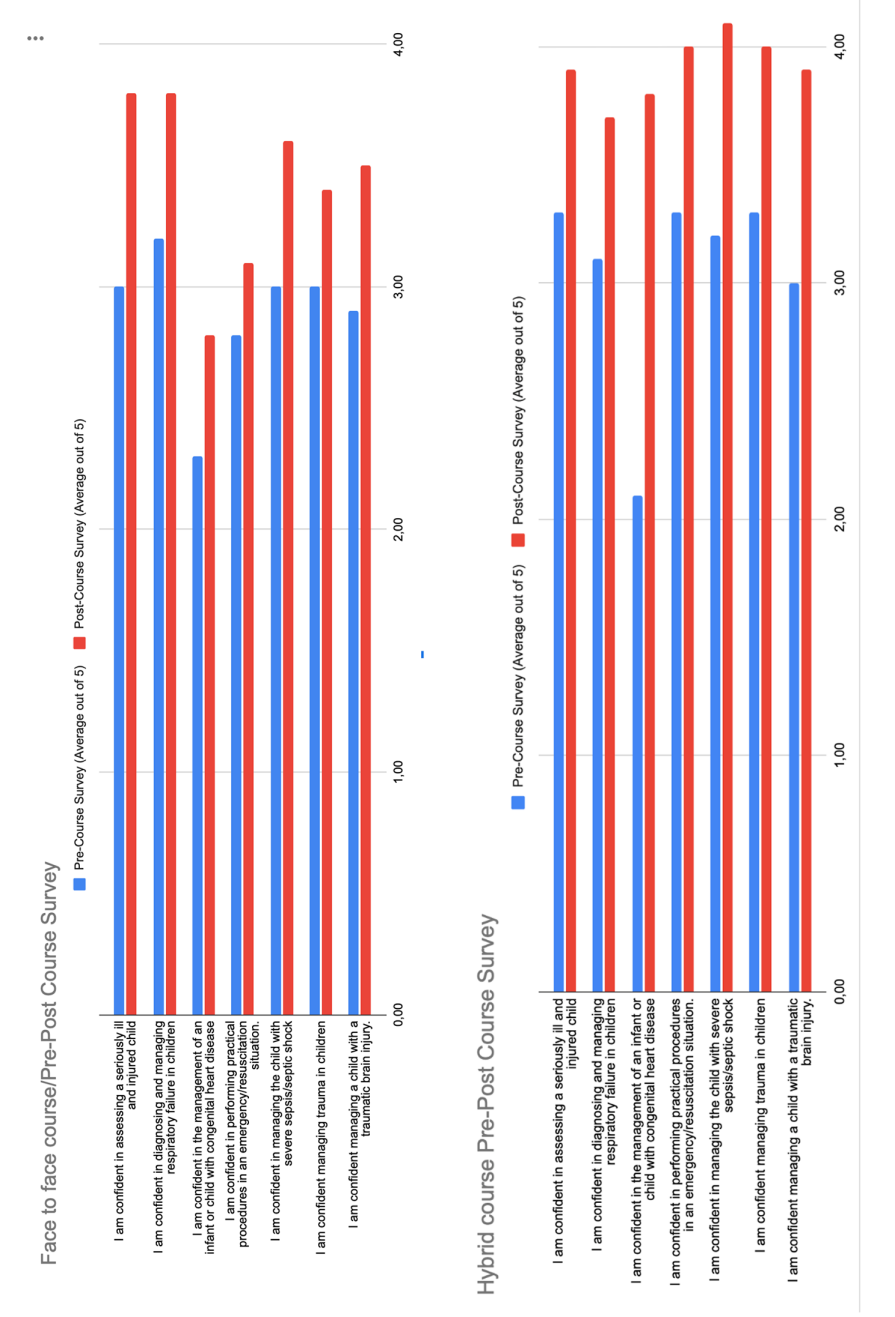
The change in Participant ‘comfort’ before and after the course: mean value of survey rating scales of face to face and hybrid course (FC = face to face course, HC = hybrid course)

The change in participant ‘comfort’ or confidence with fundamental clinical knowledge and skills due to course participation provided an understanding of the outcome of the educational process at Kirkpatrick Level 2. For the question about confidence in managing emergency airway procedures in critically- ill children, we found higher scores in the face-to-face course (Fig. 4). Conversely, we found higher rates in the hybrid groups in regard to managing congenital heart diseases, trauma, and septic shock in the post-assessment. Results between pre and post-course scores were then compared by counting the number of participants who moved from scoring < 3 (“strongly disagree”” and “disagree”) to scoring > 3 (“agree” and “strongly agree”) (Table 3).

One single question was sent to attendees six months after the course asking if the course was relevant to their recent clinical practice. 75% of the attendees responded, scoring > 3 (“strongly agree” and “agree”), considering respiratory failure and mechanical ventilation as the most relevant modules.

The engagement of the standard BASIC course was very good, many attendees reported that there was a good atmosphere and a safe learning environment. After both the standard course and the hybrid course there were two meetings for the faculty debriefing experience. From this, it seems that the hybrid format was more challenging for both attendees and instructors, especially the virtual workshops. The recorded lectures were reported as a positive aspect by attendees because they allow flexibility in terms of timing and the opportunity to view the material repeatedly. As the lectures were all in English, this was considered helpful by students who were not very confident with the English language.

The virtual workshops, run before face-to-face two days-workshops, were delivered through video conferencing and synchronous chat rooms. The faculty reported a progression in the engagement and interaction of the attendees during these synchronous virtual meetings.

## Discussion

The transition to virtual education models during the COVID-19 outbreak has been well described in the scientific literature over the last two years [6]. This course has demonstrated the flexibility and feasibility of transitioning to a hybrid model. In addition, no significant differences were found using a number of different evaluative methods between the two modes of course.

We suggest that these findings support the rationale that because the course contents are highly standardized, they are perceived by students in the same way regardless if presented face-to-face or virtually. At the end of both courses, the skills stations and simulated scenarios were highly appreciated and individually described as one of the most interesting parts of the course. The request for more time for practice is understandable, underlying the value of experiential sessions. Experiential learning entails a hands-on approach compared to more frontal instruction, making simulation a more personal and engaging way of learning. Although there was no significant difference in the pre and post-survey confidence levels reported by participants for most items, students of the face-to-face course reported a general shift in confidence compared to the hybrid course.

Conversely, the hybrid group had higher rates of self-confidence compared to the standard group, especially in emergency procedures. Maybe this is because there was a strong presence (22%) of anesthesiologists in the hybrid group, which was the only difference in the group’s composition. Probably this difference among the participants composition is also responsible about the higher pre and post assessment scores of the HC group compared with the FC (Table 2). This difference in a deeper perspective can be also responsible about the shift in pre and post assessment variability shown in Fig. 3. But despite the difference between the two starting groups, after the course in both FC and HC groups most of the attendees achieved a score > 80% correct in the post-course MCQ. This basal differentiation among groups affect the final scores, but we have to consider that anaesthesiologists, paediatricians and adult emergency physicians are all professional categories part of the target of BASIC course itself and the composition of the groups of attendees can have high variability in each course for several reasons. Possible implications of these findings have not been studied due to the small size of both groups. This comparison between FC and HC as part of a pilot experience represents a starting point for more accurate consideration for example in the group constitution and related educational offers. The possibility to study a larger sample of participants will provide a more precise statistical analysis on this topics.

To better define the difference reported for these specific items, a larger sample from a greater number of courses would be required.

The faculty reported a progression in the engagement and interaction of the attendees during synchronous virtual meetings in the hybrid format, probably due to the increasing confidence of the attendees with this approach and also with the faculty members. We used the “flipped classroom’’ approach, one of the models of virtual learning, where lectures are delivered through recordings that students view outside of class, thereby saving (virtual) in-class time for individual or group virtual practice and exercises, with the instructor present to assist and answer questions [7]. That means that our theoretical pictures required the students to prepare themselves for face-to-face practical sessions and engage with the material beforehand [7, 8]. In this sense, they were considered not as ‘homework’ but essential groundwork for productive class time [9]. Studies in different fields, such as law and psychology, suggest that distance learning may not increase benefits compared to traditional in-class instruction but are as effective [10]. This is an important point in terms of feasibility and effectiveness for designing new learning formats and also in the perspective of diffusion of specific learning content to a larger audience. Furthermore, online or blended courses allow the participation of students from all over the world, bringing together diverse people, cultures, and communities outside our physical location. This also offers the possibility to lower costs for building teaching modules and to provide more equal access to learning opportunities.

The pre-recorded lectures on the virtual platform represented an advantage for non-native English speakers since it was possible to listen to and review the theoretical lessons for a period of time sufficient to ensure adequate understanding by all the students. Unlike their face-to-face counterparts, online courses are predominantly asynchronous, where both faculty and students manage this themselves to a large extent. Asynchronous courses present an advantage to adult learners who need a flexible schedule. The diverse global faculty provided the candidates with opportunities to compare multiple perspectives from colleagues from all over the world, as the pre-recorded lessons and virtual meetings involved professionals from more than ten highly specialized training sites. Despite the progressive increase over the pandemic period to virtual and online teaching, some of the instructors noted the time-consuming aspect of using and implementing new technologies and the need for both students and faculty to be comfortable with the platform. On the other side, gaining familiarity with this virtual material can make it progressively easier to use and allowed the faculty to create a pool of lectures and recorded material that can be used repeatedly for many courses. Another challenging aspect of the hybrid course was the online workshops and simulation scenarios conducted locally but supervised and debriefed remotely using the online format. Conducting these sessions in English was challenging and highlighted the importance of having local facilitators involved in all sessions to support participants if language impeded the conduct of the session. The application of remote simulation as a practice for distance training is now increasingly widespread [11–13]. This necessarily requires a high degree of preparation and engagement on the part of the instructors [11]. However, the participants considered these experiences just as favorably as the same skill stations and simulated scenarios in the face-to-face format. In face-to-face learning, the instructors encourage the students to connect with others and share their experiences. If virtual and blended learning are delivered appropriately, the instructor can maintain this role with the same aims and scopes. This can be a challenge as it can be difficult to maintain a high attention level of students in the virtual context and manage the complexity of the learning environment.

## Conclusion

The transition to virtual education models during the COVID-19 pandemic led to the creation of a hybrid pediatric BASIC course. We report the flexibility and feasibility of transitioning to such a hybrid model without significant differences in attendees’ performances, appreciation, and self-confidence. Using this hybrid model beyond the contingency of the pandemic might improve the homogeneity of education and clinical practice in Italian PCCM. It could make core training practicable on a large scale, potentially at the national level. The importance of courses such as pediatric BASIC to countries without clear and structured training for PCCM, such as Italy, could provide the bedrock of induction into formal PCCM training and support clinicians tasked with caring for critically ill children without the bespoke training provided in other countries in the interim. It fills an institutional and academic void, which scientific societies, hospital administration teams, and licensing bodies should support.

## Electronic supplementary material

Below is the link to the electronic supplementary material.

Supplementary Material 1

## Data Availability

All data generated or analyzed during this study are included in this published article.

## List of abbreviations

PCCM: Pediatric Critical Care Medicine
ESPNIC: European Society of Paediatric and Neonatal Intensive Care
EPIC: European Paediatric Intensive Care
PICU: Pediatric Intensive Care Unit
NICU: Neonatal Intensive Care Unit
ICU: Intensive Care Unit
US: United States
MCQ: Multiple Choice Question
FC: Face-to-face Course
HC: Hybrid Course
