# Supplementary material for: Pediatric basic course goes virtual: transition from face to face to hybrid learning in pediatric critical care
Source: Ital J Pediatr. 2023 Jun 7;49:67. doi: 10.1186/s13052-023-01461-4 (PMC10246866; doi:10.1186/s13052-023-01461-4)
Supplement: Supplementary file 1 — Supplementary Material 1 [file 13052_2023_1461_MOESM1_ESM.docx]

Padova, 13th of January 2023

**Response to reviewers**

Reviewer #1: The authors proposed a comparison of a pediatric basic course based on two different scenaries (face to face and an hybrid format), in which they stated the impact on knowledge and perception in managing pediatric population.

The manuscript information has an acceptable originality, however there are some points I'd like to highlight to improve the quality of the document:

1) As one of the main differences between courses were the Access of the pre-recorded lectures, the autors could offer more information regarding those (What kind of material they offer? What was the duration of the lectures? Do the lectures were recored ad-hoc this course or it was a compillation of some other sources?

R: We thank the reviewer for this comment and for the opportunity to detail more the set of pre-recorded lectures. We provided 15 lectures covering all the most important topics in pediatric critical care of 30 min each, recorded by a pool of experts in the field who agreed to collaborate on this project. The Faculty included professionals from Australia, Europe and the US, currently teaching in standard in presence BASIC courses. All the lectures were recorded expressly for this edition of BASIC course. Each lecture provided some insights on specific subtopics according to the program of the course and some clinical cases were presented.

2) Do the authors have some statistics regarding the reviewes of this material which could indicated the "complicated topics" or the ones who attracted to the students more?

R:As listed in table 3 of the previous version (now figure 2 of the reviewed version), we collected the rate evaluation of the attendees for both face to face and hybrid courses modules. Each topic (including in some cases multiple lectures) was rated by asking the attendees to consider if the theoretical part (lectures) and the practical part (skills station, discussion) were interesting and complete. Sepsis and airway management were the most attractive in the face to face course and acute kidney injury and cardiopulmonary interactions in the hybrid course in simple rating. It is worth noting that these 4 modules were presented in both face to face and hybrid courses by the same instructors. We also collected at the end of the courses some open comments by the attendees in order to give them the possibility to detail more their difficulties or suggestions: among the attendees’ comments collected in a word cloud we found that ventilation and fluids management were perceived as important topics in pediatric critical care that deserved a deeper and longer learning module. Nephrology was reported as a complicated topic which could have a broader approach and deepening.

3) What does these final thoughts of the students indicate? Do they indicate that the experience was good or bad? Why? Please, discuss it a little bit.

R: The overall evaluation of both the face to face course and hybrid one was positive. 94% of the attendees of both courses affirmed that the material was complete, appropriate and interesting (in a likert scale 1 to 5) and that the course met the quality expectations they had. Among the suggestions we collected thanks to the open questions, the strengths are the fact that the course is run by an international faculty with the possibilities to have a direct exchange in terms of answering questions and have some practical simulation and skills stations. In all courses the attendees were asking for more time dedicated to practical skills stations and scenarios. Another important remark was the possibility to have a stratification of the attendees according to the experience in managing the critically ill child.

4) How did students engage on both courses? Do the students face some difficulties in the hybrid format? If so, how do they were managed?

R: The engagement of the standard BASIC course was very good, many attendees reported that there was a good atmosphere and a safe learning environment. After both the standard course and the hybrid course there were 2 meetings for the faculty debriefing experience. From these it results that the hybrid format was more challenging for both attendees and instructors, especially in the virtual workshops. The recorded lectures were experienced by the attendees as a positive aspect of the course, especially because they can provide flexibility in terms of timing and the opportunity to repeatedly review presented material. As the lectures were all in English this was considered a facilitation especially for those students who were not very confident with the English language.

The virtual workshops, run before the face to face 2 days-workshops, were delivered through video conferencing and synchronous chat rooms. The faculty reported a progression in the engagement and interaction of the attendees during these synchronous virtual meetings ( a total of 4), probably due to the increasing confidence of the attendees with this approach and also with the faculty members. This “flipped classroom'', as one of the models, can deliver lectures through recordings that students view outside of class, thereby saving in-class time for individual or group virtual practice and exercises, with the instructor present to assist and answer questions.

Despite the progressive increase over the pandemic to virtual and online teaching, some of the instructors also noted the time-consuming aspect of the use and implementation of new technologies and the need for students and also for the faculty to be comfortable with the platform and these new virtual activities.

5) How did the instructor serve as a facilitator in this process on both formats? This aspect has not been integrated well into the manuscript. More information on this needs to be included.

R: In a face-to-face learning course as the instructors and the attendees are in the same time and place, the content and learning material are taught in person, allowing for a live interaction between the student and the instructor but also encouraging students to connect with others and learn socializing and sharing their experiences with different people. In virtual and blended learning this role is kept by the instructor with the same aims and scopes. The challenging aspect is represented by the fact that it can be more difficult to maintain high the attention level of the student in a virtual context and that it can be difficult to manage the complexity of the learning environment.

The instructor serves as facilitator of this process both in lectures and in the simulation and skill station part. In the practical part as we are dealing with adult learners, it is mandatory to strengthen competencies and reinforce good professional behaviors supporting them towards achieving concrete learning objectives. The facilitator needs to continuously adapt his/hers educational psychology and teaching strategies to the attendees obtaining feedback through non-judgmental discussion and learner reflection, according to simulation teaching principles.

6) Which are the differences between these pre-recorded lectures vs the face to face? -Theorically speaking and based on some learning models or pedagogical aspects, which will be the principal points to develop in the discussion section.

R: Unlike their face-to-face counterparts, online courses are predominantly asynchronous, where both faculty and students manage this themselves to a large extent. Asynchronous courses present an advantage to adult learners who need a flexible schedule. Moreover, asynchronous courses give the possibility to students to pause the recording while they take notes or absorb the information and to review the course lectures and the materials multiple times. This has the potential to develop students' critical thinking and reflection, which deepens their understanding of the content (Martin & Bolliger, [2018](https://www.ncbi.nlm.nih.gov/pmc/articles/PMC9111674/#bjet13190-bib-0049)). Pre recorded lectures can also be interactive as the face to face courses, that is having some interactive elements or polls to answer or introducing learning activities related to communication, collaboration, discovery and search for information, active listening, and activities that require the direct involvement of other students. Diverse communication skills are primary in online learning. While face-to-face we have more lecture- and dialogue-based learning exercises, the advantage of having diversely communicated course content is that students may use multidimensional materials and also learn digital literacy and netiquette which is definitely important nowadays. As we planned in our course, the pre-recorded lectures were considered as part of a [flipped classroom](https://fetliu.net/blog/flipped-learning/) approach. That means that our theoretical pictures were the requirement for the students to prepare themselves for face-to-face practical sessions and engage with the material beforehand. In this sense they were considered not as ‘homework’ but essential groundwork for productive class time (Salas Pilco et al, 2022). Some studies in different learning fields as law or psychological studies suggest that distance learning may not present increased benefits compared to traditional in-class instructions, but that it is as effective (Bahnson et al, 2017). This is an important point in terms of feasibility and effectiveness for designing new learning formats and also in the perspective of diffusion of specific learning content to a large audience. Online or blended courses allow the participation of students from all over the world, bringing together diverse people, cultures, communities, outside of our physical location. This also offers the possibility to lower costs for building teaching modules and to offer a more equal access to learning opportunities.

7) I think the discussion section must be improved and contrast their results with some other studies from the same field or similar. In this suggested references the authors can reach some similar approaches on several areas or in medical/health sciences ones.

Salas-Pilco, S. Z., Yang, Y., & Zhang, Z. (2022). Student engagement in online learning in Latin American higher education during the COVID-19 pandemic: A systematic review. British journal of educational technology : journal of the Council for Educational Technology, 53(3), 593-619. <https://doi.org/10.1111/bjet.13190>

R: We thank the reviewer for the reference suggestion. We added in the discussion some reports with similar experiences.

8) The authors presented the student perspective but it would be enriching if they could describe the instructor's perspective as well.

R: Many thanks for this point, a paragraph has been added in the result section.

9) I would suggest the authors improve the design and presentation of their tables and data, as they are not easy to follow.

R: We thank the suggestion of the reviewer, we added some figures and improved the design.

10) Finally, there are several grammatical errors and unclear sentences in the manuscript. Check some typing errors as Page 4, line 57; Page 5, line 30, 35 (doublé "") and others.

R: Many thanks for this input. We modified all the listed sentences and paragraphs in order to male them clearer. Here the corrections suggested by the reviewer but many more has been integrated in the text as this has been reviewed again by an English native author.

Page 3, line 3 we added the adjective *“Pediatric” Critical Care Medicine (PCCM)*

Page 3, line 16.17: *rephrased*

Page 4, line 57: *in* collaboration

Page 5, line 30

Reviewer #2: Authors have compared the benefit of a face to face Pediatric Basic course with an hybrid one. There are some hints that both courses increased confidence of participants in treating severely ill children.

The introduction is too long . The first 3 pages and an half need to be shortened.

R: We thank the reviewer for this point, we shortened the introduction section.

Page 5. Line 41-Page 6, line 1. This paragraph should be moved to the method section.

Methods. How were participants selected?

R: All the participants enrolled in the course spontaneously, the course was promoted and advertised by the University of Udine, the BASIC course website, ESPNIC website. An informed consent has been signed before the registration in order to collect the agreement to be surveyed.

Page 6, line 21. Pre and post-assessment scores (MCQ) should be adequately reported.

R: mean value of pre and post assessment scores are reported in table 2 for both standard and hybrid format. Two figures were also added to compare pre and post assessment scores of FC and HC groups.

Results. It is of interest to know whether learning and confidence varied among participant with different specialization.

R: We thank the reviewer for this input but we decided not to perform this analysis as the small number of each subspecialty group would likely make the statistical analysis not reliable. Nevertheless, we consider this approach extremely interesting and it will be possible to carry out this analysis following the dissemination of the course and the possibility of repeating the observation on different professional groups in order to evaluate the impact of this course more correctly.

Page 7 , line 41. A new table reporting results of pre and post-course MCQ tests is warranted.

R: Two figures were added to compare pre and post assessment scores of FC and HC groups.

Conclusions are not supported by results and should be rewritten sticking to findings. It is unclear why the hybrid model beyond the contingency of the pandemic would undoubtedly improve education and clinical practice. Another point is what indicators show that the importance of pediatric BASIC courses in countries like Italy is evident.

R: Result section, as well as discussion and conclusion section were reviewed and many amendments has been done in order to improve them and to argument more these points.
